# New presentation of CLIFAHDD syndrome with a novel variant in *NALCN* gene: A report of a rare case

**DOI:** 10.1002/ccr3.7647

**Published:** 2023-07-17

**Authors:** Bita Hashemi, Richard J. Huntsman, Huan Li, Dapeng Zhang, Yanwei Xi

**Affiliations:** ^1^ Department of Pediatrics, Division of Medical Genetics University of Saskatchewan Saskatoon Saskatchewan Canada; ^2^ Department of Pediatrics, Division of Neurology University of Saskatchewan Saskatoon Saskatchewan Canada; ^3^ Department of Biology, College of Arts and Sciences Saint Louis University Saint Louis Missouri USA; ^4^ Program of Bioinformatics and Computational Biology, College of Arts and Sciences Saint Louis University Saint Louis Missouri USA; ^5^ Department of Pathology and Laboratory Medicine, Genomics Laboratory University of Saskatchewan Saskatoon Saskatchewan Canada

**Keywords:** CLIFAHDD syndrome, distal arthrogryposis, NALCN gene

## Abstract

**Key Clinical Message:**

Congenital Contractures of Limbs and Face, Hypotonia, and Developmental Delay (CLIFAHDD) syndrome is a recently described type of distal arthrogryposis which unlike other subtypes is associated with developmental delay and various neurologic presentation. Epilepsy and ataxia have been reported. We add paroxysmal dyskinesia to the clinical spectrum. Understanding the molecular mechanism can help developing targeted therapy in future.

**Abstract:**

This study resulted in identification of a novel variant in *NALCN* gene leading to autosomal dominant CLIFAHDD syndrome. Our patient presented with a form of nonepileptic paroxysmal dyskinesia. This is a new phenotype that has not been described previously.

## Graphical abstract

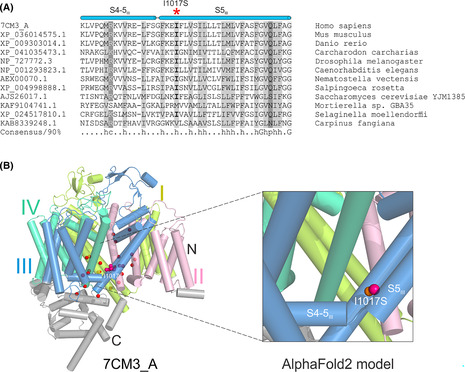

## 1 INTRODUCTION

The NALCN channelosome is a conserved multiprotein cation channel expressed predominantly in neurons. NALCN forms the channel pore of the complex and by way of a persistent sodium leak, maintains the membrane resting potential.^1^ Both monoallelic and biallelic pathogenic variants have been described and lead to different clinical phenotypes with some overlapping features.^2^ CLIFAHDD syndrome is the result of heterozygous gain of function variants.^2^ Patients present with distal arthrogryposis in a pattern similar to Freeman Sheldon syndrome at birth; however, they have additional findings including hypotonia and respiratory abnormalities and will later manifest variable degrees of global developmental delay.^2^ Some patients have epileptic seizures.^3^ Brain magnetic resonance imaging (MRI) may show cerebellar or corpus callosum abnormalities.^4^ This report presents a patient with symptoms suggestive of CLIFAHDD syndrome and a novel variant in *NALCN* gene. Our patient also has a neurologic finding that has not been described previously.

## 2 MATERIALS AND METHODS

To identify a possible underlying genetic cause for the patient's presentation, genomic DNA was obtained from blood and an Arthrogryposis gene panel (14 genes: *CHST14, ECEL1, FBN2, MYBPC1, MYH3, MYH8, MYL11, NALCN, PIEZO2, TNNI2, TNNT3, TPM2, MAGEL2, PNKD*) was arranged through a private laboratory. Next‐generation sequencing technology was used to sequence the coding regions of the targeted genes plus 10 bases of noncoding DNA flanking exonic/intronic regions. >98% of target bases were covered at >20X and mean coverage of target bases were >100X. *NALCN* variant was confirmed via Sanger sequencing in proband. This variant was submitted to ClinVar (Accession: SCV002576520). Consent was obtained from family for publication of clinical data and pictures.

## 3 CASE PRESENTATION

The proband is a 14 years old male and the only child of a non‐consanguineous couple of Cree/Saulteaux/Irish descent. Family history is unremarkable for any neuromuscular or congenital anomalies. Parents were separated shortly after his birth.

He was the product of an unremarkable pregnancy to a healthy 17‐year‐old G1P0 mother. He was born at term via vacuum assisted vaginal delivery. His Apgar scores were 9 and 9 at 1 and 5 min, respectively. His birth weight was 3390 gr (25‐50th), length 47.5 cm (10‐25th) and head circumference 35 cm (mean). He was noted to have bilateral club feet and camptodactyly. A karyotype, chromosomal microarray, and CK were normal. *TPM2* molecular testing came back normal. He had corrective surgeries for his club feet and camptodactyly.

Initially, he had developmental delay limited to speech with first words spoken at around 16 months of age. He subsequently experienced some regression with his motor developmental milestones around 7 years of age when he developed abnormal paroxysmal events that initially consisted of him having sudden onset motor arrest, staring and losing balance. His mother felt he was scared during these events. They lasted about 1 min with no post ictal phase. A 24‐h electroencephalogram (EEG) telemetry was performed which showed normal background activity during wakefulness and sleep and no epileptiform discharges. One event was captured with no associated electrographic changes, and they were therefore deemed to be nonepileptic.

These events became more frequent and longer in duration over time. They were triggered by states of heightened emotion in particular excitation, stress, and physical exertion. A few minutes after the trigger, he will indicate he is tired, sit down, and then slump over to one side (usually the left). He is unable to move but consciousness appears spared as he remains aware of his surrounding and can track visually. At times dystonic grimacing of the face and tongue protrusion would occur. With longer events, a fine tremulous activity of the extremities is noted. These events are now occurring several times per month and last anywhere from 3 to 20 min in duration. Two recent prolonged EEG telemetry performed recently failed to capture any events but did show occasional interictal bifrontal sharp waves indicting a tendency toward seizure arising from either frontal lobe. Despite these changes on the EEG, these events are still felt by pediatric neurology to be atypical for epileptic seizure and more in keeping with paroxysmal dyskinesia. His cardiology workup was unremarkable. He has obstructive sleep apnea with otherwise no respiratory symptoms. His recent MRI showed only mild cerebellar atrophy.

**FIGURE 1:**
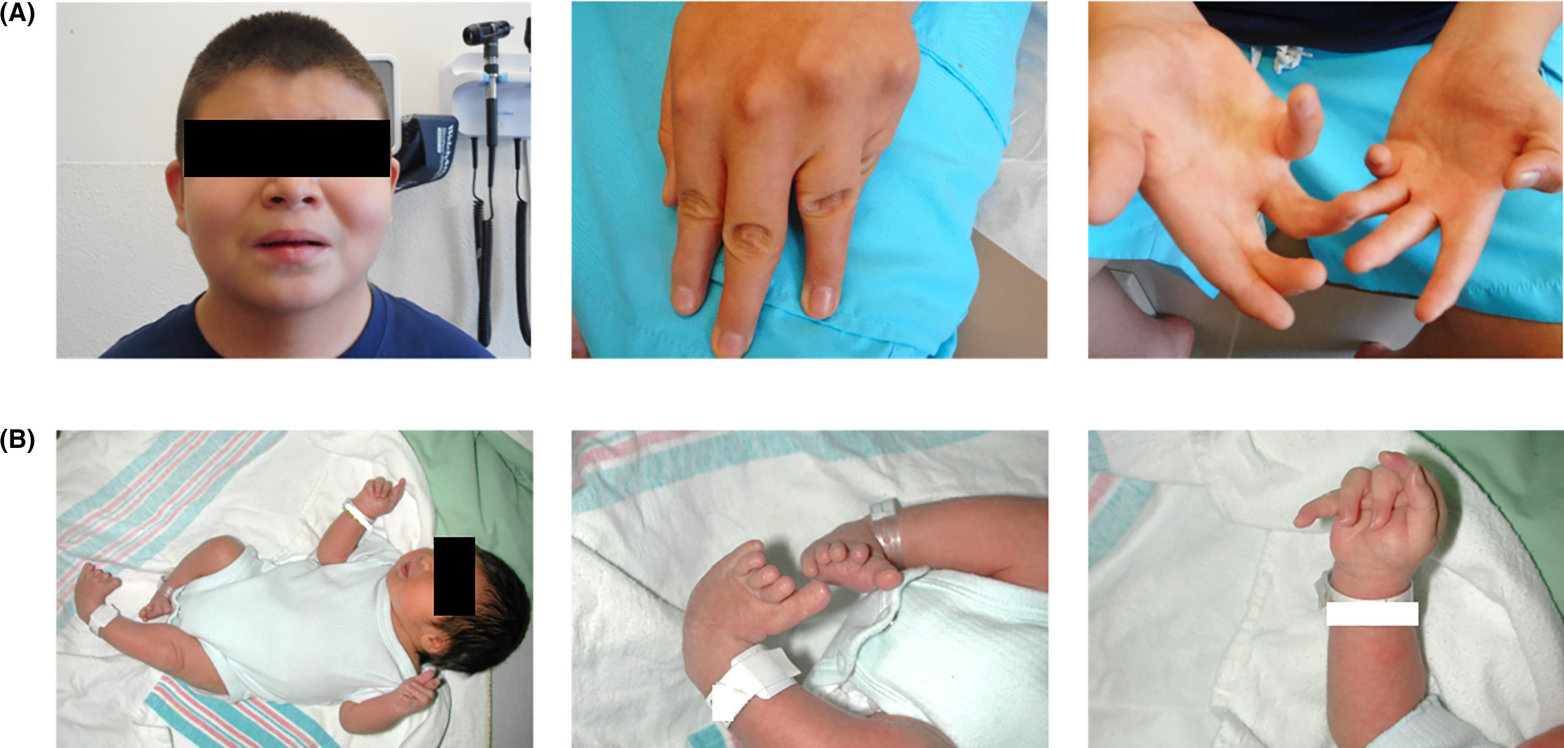
(A) Index patient at the age of 14 years old – minor dysmorphic features in the examination: downslanting palpebral fissures, wide nasal bridge, prominent nasolabial folds, downturned corners of the mouth; bilateral camptodactyly with absence of distal interphalangeal creases and ulnar deviation of hands. (B) bilateral clubfeet and camptodactyly at birth.

His physical examination at the age of 14 years old showed growth parameters within normal limits except for truncal obesity. He had a masklike facies without obvious whistling mouth appearance. His neck was short with a buffalo hump. He had bilateral camptodactyly with ulnar deviation of hands and tapering of fingers. His club feet were corrected by surgery. Otherwise, there was no restriction to joint movements, and neurologic examination was unremarkable (Figure 1A,B).

## 4 RESULTS

Due to these nonepileptic events, molecular testing for episodic ataxia panel (*CACNA1A, CACNB4, KCNA1, PNKD, PRRT2, SLAC1A3, VAMP1*) was requested, and this came back normal. Considering the presence of distal arthrogryposis and global developmental delay, an arthrogryposis gene panel was arranged which showed a sequence variant in *NALCN* gene designated as c.3050T > G, which is predicted to result in the amino acid substitution (p.Ile1017Ser; NM_052867.2). This variant was not maternally inherited, and father was not available for testing. This variant was initially classified as unknown clinical significance by the providing laboratory. With further literature research, we classified this variant as likely pathogenic based on ACMG 2015 guideline (PM1, PM2, PM5, and PP3).

**FIGURE 2:**
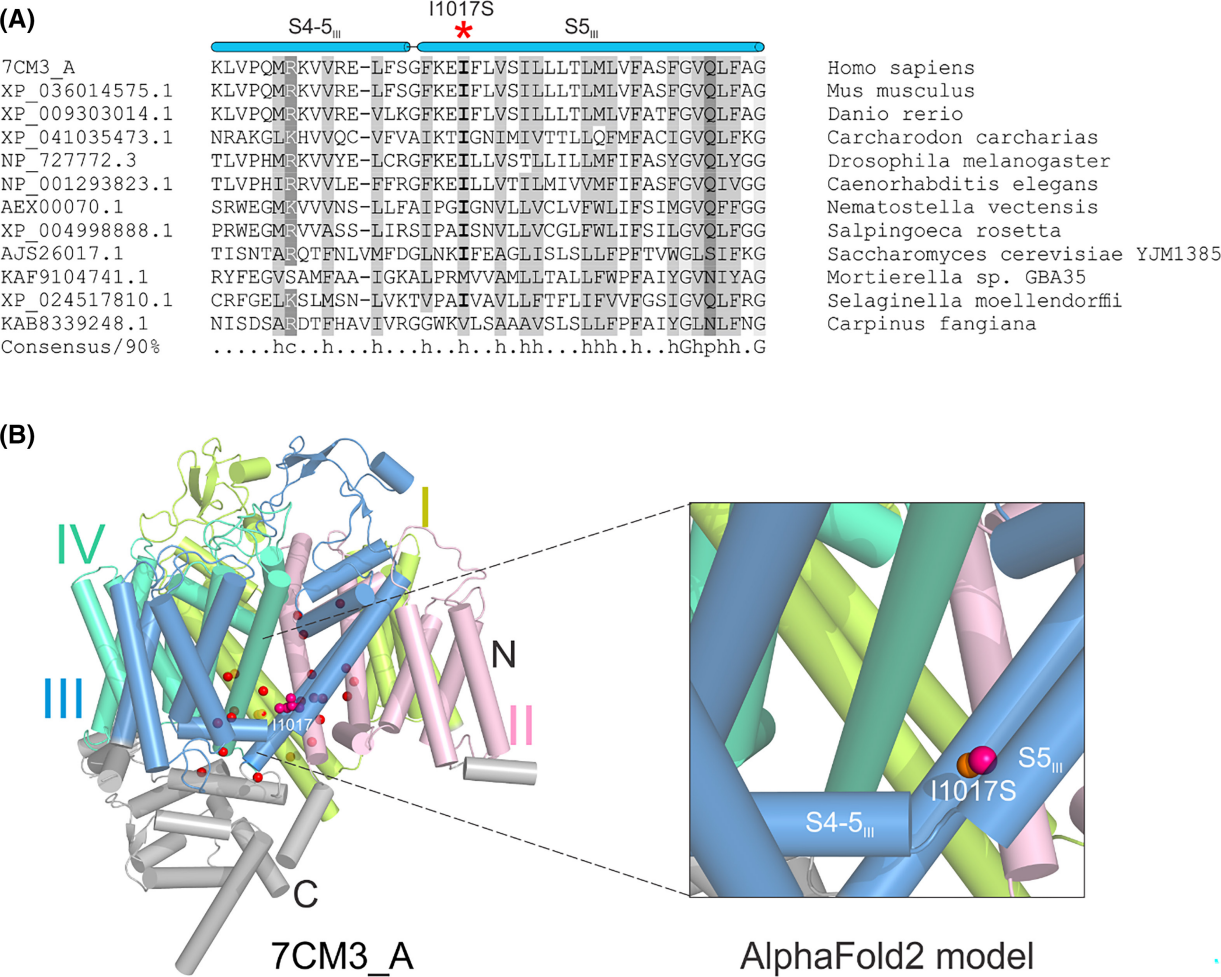
(A) Multiple sequence alignment of homologous NALCN fragments around Ile1017. Each sequence is labeled by its NCBI accession number and species name. The position of Ile1017 was highlighted by a red asterisk. S4‐5_III_, the linker helix between S4 and S5 in DIII; S5_III_, the S5 in DIII. (B) Structural View of human NALCN (7CM3_A, left panel) and the AlphaFold2 model of the mutant NALCN with Ile1017Ser (right panel). DI was shown in limon, DII in light pink, DIII in sky blue, and DIV in lime green. Twenty‐five residues whose missense mutations have been reported to be associated with CLIFAHDD are showed as spheres. For 24 of the 25 residues, only their alpha carbon atoms were shown and highlighted in red. While for Ile1017, both the carbon atoms and side chain are shown and highlighted in hot pink. The substituted serine on the same position has a sulfur atom shown in orange in the right panel.

NALCN has four homologous domains (DI‐DIV) to form the transmembrane region. Each domain has six transmembrane helixes (TMs, S1‐S6), of which the first four TMs (S1–S4) constitute the voltage‐sensing domain (VSD) while S5 and S6 are involved in the formation of ion‐conducting pore.^5^ The newly identified mutation Ile1017Ser is located on the S5 TM of the domain III (DIII). To understand the potential effect of Ile1017Ser in NALCN channelopathy, homologous sequences of DIII was compared within a wide range of species. The multiple sequence alignment reveals that Ile1017 is conserved among animals, and also conserved for being hydrophobic in fungal and plant species (Figure 2A). The high conservation across a long evolutionary time suggests a purifying selection of functional constraint on this position. Therefore, the polar property introduced by Ile1017Ser might have disrupted such constraint. Various *NALCN* mutations have been found associated with human CLIFAHDD (Figure 2B, left panel).^1^, ^2^, ^5^, ^6^ Interestingly, the majority of these missense mutations, including Ile1017Ser found in this study, are located on the S5 and S6 TMs that^7^ form the ion‐conducting pore. Experimental studies tested the electrophysiological properties of many NALCN mutants revealed that almost all mutations, including an Ile1017Thr, a comparable mutation as our Ile1017Ser, resulted in gain‐of‐function phenotypes.^8^ In other words, these mutations would shift NALCN in patients to a more active state, for example, generating greater Na^+^ leak currents. Given the comparable property change introduced by both Ile1017Thr and the newly identified Ile1017Ser, we propose that the Ile1017Ser mutation would display a similar gain‐of‐function phenotype due to the switch of the property from hydrophobic to polar.

## 5 DISCUSSION

In this paper, we report CLIFAHDD syndrome in a 14‐year‐old male patient with a novel variant in *NALCN* gene. Although seizures and ataxia have been previously reported in CLIFAHDD syndrome, our patient also has recurrent episodes of paroxysmal dyskinesia contributing to his neurodevelopmental regression which to our knowledge have not been reported previously.

Currently, all reported *NALCN* variants are de novo located in or near the S5 or S6 segments. The novel variant in our patient is located on S5 TM of the domain III. Comparative sequence analysis and in silico modeling indicate the p.Ile1017Ser change could result in an alteration from hydrophobic to polar property. Functional studies in a comparable mutation at the same location have shown a gain‐of function; thus, we propose this variant being responsible for patient's phenotype.

In summary, here we describe a novel *NACLN* variant associated with CLIFAHDD syndrome and a new neurologic finding which will expand the clinical spectrum of NALCN‐related disorders.

## AUTHOR CONTRIBUTIONS

**Bita Hashemi:** Writing – original draft; writing – review and editing. **Richard J. Huntsman:** Writing – review and editing. **Huan Li:** Software; validation; visualization. **Dapeng Zhang:** Data curation; software; visualization. **Yanwei Xi:** Validation; writing – original draft; writing – review and editing.

## FUNDING INFORMATION

No specific funding was required for this study.

## CONFLICT OF INTEREST STATEMENT

The authors declare no competing interest.

## PATIENT CONSENT STATEMENT

Written informed consent was provided by the family for publishing clinical data and pictures.

## Data Availability

The specific variant has been submitted to Variant Validator and submitted in supplement material.
